# Supplementary material for: Pseudomonas aeruginosa pqs Quorum Sensing Mediates Interaction with Mycobacterium abscessus In Vitro
Source: Microorganisms. 2025 Jan 8;13(1):116. doi: 10.3390/microorganisms13010116 (PMC11768086; doi:10.3390/microorganisms13010116)
Supplement: Supplementary file 1 [file microorganisms-13-00116-s001.zip › microorganisms-3367526-supplementary.pdf]

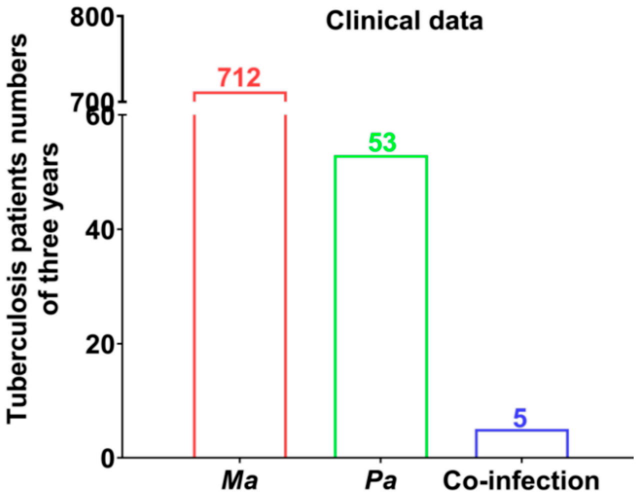

Supplementary Figure S1. The number of clinical patients infected with *M. abscessus*, *P. aeruginosa*, and co-infected with both among tuberculosis patients in Shenzhen Third People's Hospital over three years

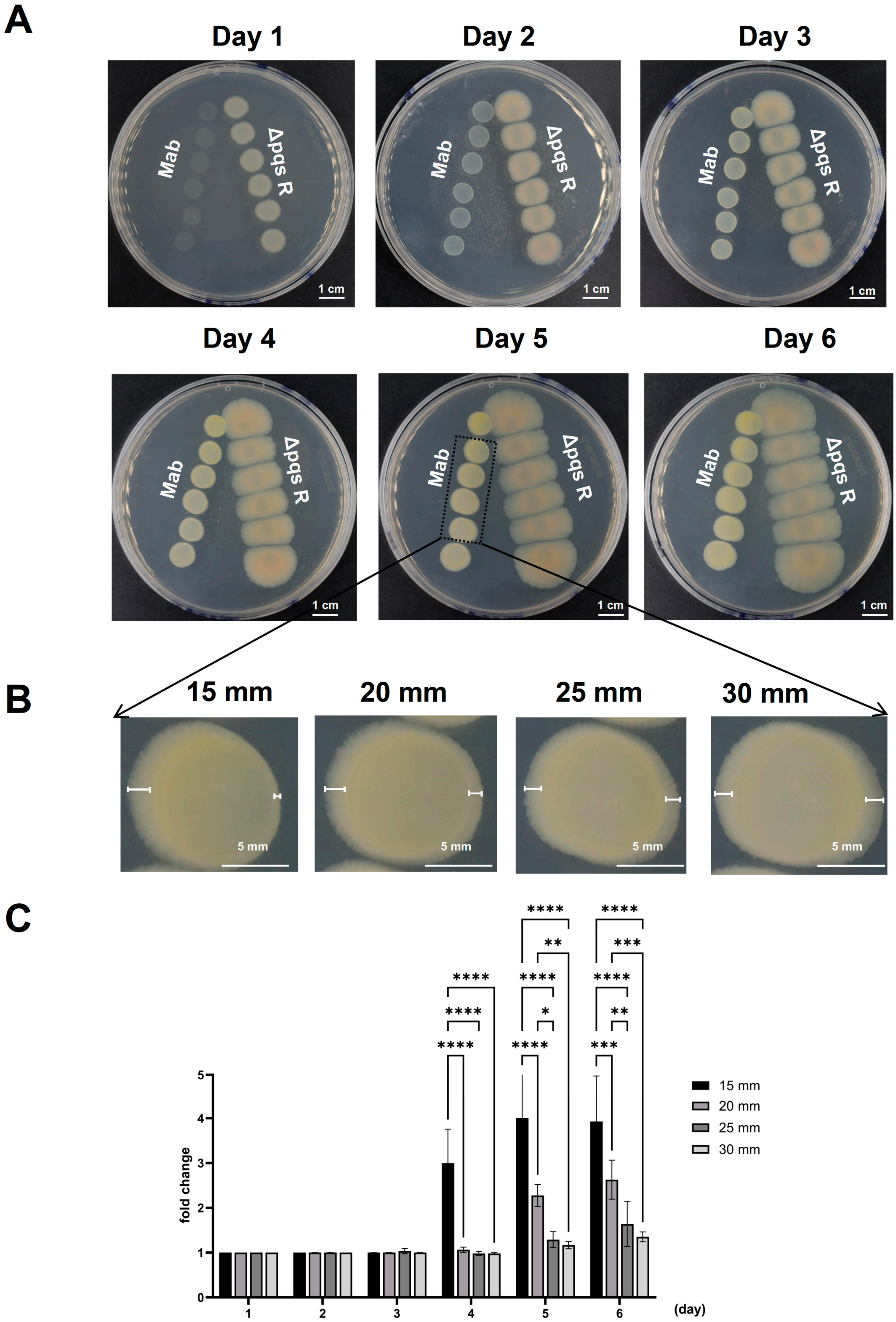

Supplementary Figure S2. Interaction between *M. abscessus* and Δ*pqsR P. aeruginosa*. (A) Pairwise proximity assays showing the interaction between *M. abscessus* and Δ*pqsR P. aeruginosa* (Pa PAO1) at varying distances (15 mm, 20 mm, 25 mm, 30 mm). **(B)** Magnified view of *M. abscessus* colonies with the measurement

method indicated. **(C)** Quantitative analysis of *M. abscessus* displacement in relation to the distance from Δ*pqsR P. aeruginosa*. Data are expressed as Mean ± SD (n=3). * $p < 0.05$, ** $p < 0.01$, *** $p < 0.001$, **** $p < 0.0001$.

Supplementary Table S1: Comparison of differentially expressed proteins in Mab+PQS group versus Mab group in heatmap

	Uniprot ID	Protein name	Gene name	Log2 FoldChange	P-value
1	B1MAI1	Prokaryotic ubiquitin-like protein Pup	pup	0.422	3.181E-02
2	B1MAR2	Glyoxalase-like domain	MAB_2252	0.306	2.894E-02
3	B1MB87	Urease accessory protein UreG	ureG	0.285	3.877E-02
4	B1MC24	Probable manganese transport transmembrane protein	MAB_2716c	0.325	1.102E-02
5	B1MC25	CBS domain-containing protein	MAB_2717c	0.530	5.892E-03
6	B1MCX2	VOC domain-containing protein	MAB_3015	0.286	4.359E-02
7	B1MCY3	DUF3039 domain-containing protein	MAB_3026c	0.454	3.868E-02
8	B1MDS1	ABC1 atypical kinase-like domain-containing protein	MAB_3314	0.403	2.592E-02
9	B1ME04	HTH tetR-type domain-containing protein	MAB_3397	0.451	1.008E-02
10	B1MEY7	Hydrolase, alpha/beta fold LipV	MAB_3517	0.323	3.975E-02
11	B1MEZ2	assimilatory sulfite reductase (ferredoxin)	MAB_3522c	0.358	1.695E-02
12	B1MFH6	Probable cytochrome P450	MAB_0276	0.332	1.361E-02
13	B1MFJ9	Transcriptional regulator, TetR family	MAB_0300c	0.305	1.179E-02
14	B1MFV8	Transcriptional regulator WhiB	whiB	0.797	1.284E-02
15	B1MFY3	Pseudouridine synthase	MAB_3654c	0.406	3.068E-02
16	B1MG74	Uncharacterized protein	MAB_3745c	0.410	3.968E-02
17	B1MG75	Uncharacterized protein	MAB_3746c	0.361	1.587E-02
18	B1MG76	ESX-1 secretion-associated protein	MAB_3747c	0.371	1.152E-02
19	B1MGC3	Large ribosomal subunit protein uL15	rplO	0.308	1.933E-02
20	B1MH92	Large ribosomal subunit protein bL33B	rpmG2	0.279	2.911E-02
21	B1MHE3	Phosphate transporter	MAB_3950c	0.376	8.470E-05
22	B1MIE1	Transcriptional regulator, TetR family	MAB_4089c	0.394	1.219E-02
23	B1MIS0	Possible thiamine biosynthesis oxidoreductase ThiO	MAB_4218c	0.319	1.567E-02
24	B1MJN1	WXG100 family type VII secretion target	MAB_4316	0.482	1.776E-03
25	B1MJR9	NADH dehydrogenase/NAD(P)H nitroreductase	MAB_4354	0.425	4.139E-02

	Uniprot ID	Protein name	Gene name	Log2 FoldChange	P-value
26	B1MKP2	Histidine kinase	MAB_1246c	0.405	1.115E-02
27	B1ML30	Putative S-adenosyl-L-methionine-dependent methyltransferase	MAB_4606c	0.306	4.193E-02
28	B1MLH9	Probable catechol-o-methyltransferase	MAB_1318c	0.278	3.276E-02
29	B1MMK1	Pyruvate dehydrogenase E1 component alpha subunit	MAB_4918c	0.398	4.924E-02
30	B1MMS5	Hypothetical regulatory protein, TetR family	MAB_1552	0.359	3.215E-02
31	B1MNS8	Uncharacterized protein	MAB_1896c	1.178	3.176E-02
32	B1MP80	Probable ferredoxin	MAB_2049c	0.383	4.806E-02
33	B1MBV0	TM2 domain-containing protein	MAB_2641c	-0.420	1.598E-02
34	B1MC94	Secreted protein	MAB_2786	-0.271	3.774E-02
35	B1MCA8	MspA protein	MAB_2800	-0.613	6.532E-03
36	B1MDC7	1-deoxy-D-xylulose 5-phosphate reductoisomerase	dxr	-1.370	4.739E-02
37	B1MDV5	Protease	MAB_3348	-0.429	2.890E-02
38	B1MDW2	Secreted hydrolase	MAB_3355	-0.319	4.510E-02
39	B1MEW6	Uncharacterized protein	MAB_3496	-0.615	6.869E-03
40	B1MG96	Sensor domain-containing protein	MAB_3767c	-0.298	4.423E-02
41	B1MGJ2	Activator of Hsp90 ATPase homologue 1-like C-terminal domain-containing protein	MAB_3863	-0.339	4.956E-02
42	B1MGJ5	Amino acid permease	MAB_3866c	-0.364	1.350E-02
43	B1MK79	Hypothetical porin	MAB_1081	-0.438	3.143E-02
44	B1MKV2	Haemophore haem-binding domain-containing protein	MAB_4528c	-0.713	2.410E-02
45	B1MNR7	Lipoprotein	MAB_1885	-0.348	4.310E-02
